# Supplementary material for: Accumulation of Metals in the Environment and Grazing Livestock near A Mongolian Mining Area
Source: Toxics. 2022 Dec 10;10(12):773. doi: 10.3390/toxics10120773 (PMC9783985; doi:10.3390/toxics10120773)
Supplement: Supplementary file 1 [file toxics-10-00773-s001.zip › toxics-2023491-supplementary.pdf]

Supplementary Table S1 Metal concentration in this study in four areas (mg/kg dry weight). * means significant difference (p < 0.05) from all other areas, as assessed by the Steel-Dwass test. The number of samples in each area is indicated in parentheses.

		Cr	Mn	Cu	Zn	As	Se	Cd	Pb
Dornogovi AE	median	10.5	237.2	5.3	28.8	4.9	10.7	0.053	7.1
(9) (mg/kg)	range	2.5 – 25.9	47.4 – 302.7	1.4 – 9.6	10.3 – 46.7	2.0 – 9.3	5.0 – 18.0	0.021 – 0.075	2.7 – 10.5
Dornogovi UB	median	7.5	127.5	3.7	12.2	2.7	5.1	0.032	4.4
(8) (mg/kg)	range	2.9 – 10.6	53.4 – 160.5	1.9 – 6.3	5.2 – 18.8	1.2 – 4.1	3.1 – 8.4	0.014 – 0.040	1.7 – 5.4
Dornogovi ZB	median	11.4	256.5	6.6	19.3	3.9	9.6	0.038	6.2
(23) (mg/kg)	range	5.1 – 120.6	86.6 – 3504.0	2.6 – 64.7	8.2 – 209.1	2.1 – 48.2	4.3 – 86.5	0.016 – 0.44	2.4 – 76.5
Tuv Aimag	median	26.5 *	377.8	18.1 *	34.5	8.8	9.3	0.069	7.5
(15) (mg/kg)	range	7.5 – 51.7	86.5 – 922.1	5.6 – 40.9	10.1 – 57.2	3.5 – 34.9	2.8 – 12.9	0.014 – 0.37	2.0 – 12.0

Supplementary Table S2 The normal range of metals in livestock. The normal range of metal concentration in cattle, goat, and sheep is described. All values have been published [29] and are expressed here in mg/kg wet weight. As mentioned, this research used dry weight for heavy metal quantification. 3.5-4.0 times concentration (wet weight) can be estimated to approximate that of dry weight for most tissues [29]. In discussion, we simply multiply by 4 times the described concentration for comparison between our study and this table.

	Cattle		Goat		Sheep	
	liver	kidney	liver	kidney	liver	kidney
Cr	0.04–3.80	0.05–6.20			0.5–1.5	
Mn	2–6	1–2	2.0–6.0	1.0–3.0	2.0–4.5	0.8–2.5
Cu	25–100	4–6	25–150	3.0–6.0	25–100	4.0–5.5
Zn	25–100	18–25	25–120	15–30	30–75	20–35
As	0.02–0.13	0.02–0.20	< 0.0018–0.006	< 0.0015–0.007	0.02–0.2	0.02–0.3
Se	0.25–0.50	1.00–1.50	0.25–1.20	0.60–1.50	0.25–1.50	0.10–3.00
Cd	0.01–0.50	0.01–0.50	< 0.20	< 0.50	0.01–1.5	0.01–0.5
Pb	0.04–0.50	0.04–1.00	< 2	< 2	0.036–0.80	0.04–0.80

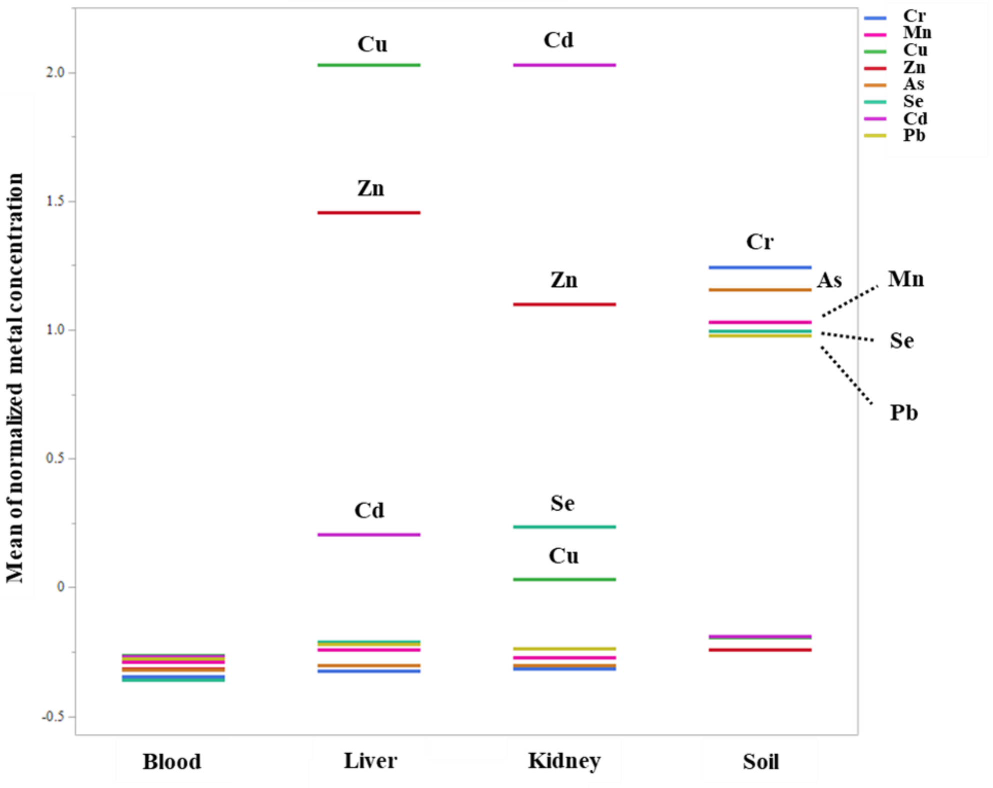

Supplementary Figure S1. Metal concentration in all the samples. Figure shows the mean of normalized metal concentration in blood, liver, kidney and soil. Each bar indicates the mean concentration of each metal in each sample type.
